# Exploring intergenerational links and genetic correlates of metacognitive beliefs: A systematic review and meta-analysis

**DOI:** 10.3389/fpsyt.2025.1674793

**Published:** 2025-11-07

**Authors:** Stefano De Francesco, Corrado Fagnani, Emanuela Medda, Sara Palmieri, Giovanni Mansueto, Giacomo De Munari, Gabriele Caselli, Simona Scaini

**Affiliations:** 1 Sigmund Freud PrivatUniversität, Fakultät für Psychologie, Vienna, Austria; 2 Centre for Behavioral Sciences and Mental Health, Italian Twin Registry, Istituto Superiore di Sanità, Rome, Italy; 3 Child and Youth Lab, Department of Psychology, Sigmund Freud University, Milan, Italy; 4 Cognitive Psychotherapy School and Research Center, Studi Cognitivi, Milan, Italy; 5 Department of Health Sciences, University of Florence, Florence, Italy; 6 School of Applied Sciences, London South Bank University, London, United Kingdom; 7 Innovation in Psychotherapy Efficiency Research Lab, Department of Psychology, Sigmund Freud University, Milan, Italy; 8 InTherapy, Gruppo Studi Cognitivi, Milan, Italy

**Keywords:** metacognitive beliefs, S-REF model, intergenerational transmission, transgenerational patterns, meta-analysis

## Abstract

**Background:**

Metacognitive beliefs, as proposed in the Self-Regulatory Executive Function (S-REF) model, are considered to play a central role in the development and maintenance of psychological disorders; however the intergenerational dimension of these beliefs remains poorly understood. Existing studies suggest potential associations between parental and offspring metacognitive beliefs, while preliminary genetic evidence indicates that some domains may be more strongly influenced by biological predispositions.

**Methods:**

A systematic search of PubMed, EBSCOhost, and SCOPUS was conducted between January and April 2025, examining studies assessing the association between parental and offspring metacognitive beliefs, as described in the S-REF model, and studies exploring links with genotype. Effect sizes were pooled for domains assessed in at least three studies, and moderator analyses considered age, study quality, and the number of covariates included.

**Results:**

Nine studies met inclusion criteria, eight focusing on parent–child associations and one on genotype. Meta-analytic results indicated small-to-moderate associations for positive metacognitive beliefs (r = .24) and negative beliefs about danger and uncontrollability of thoughts (r = .17), whereas Cognitive self consciousness did not show significant associations. Limited molecular genetic evidence suggested that Cognitive confidence and Need to control thoughts may be more strongly linked to biological predisposition. Heterogeneity was observed across studies, and moderator analyses did not reveal significant effects.

**Conclusion:**

The intergenerational dimension of metacognitive beliefs is an underexplored area with heterogeneous findings. Associations between parental and offspring beliefs are evident, particularly for Positive and Negative metacognitive beliefs, while some domains may reflect biological influences. Future research should employ longitudinal designs, comprehensive assessment across all metacognitive domains, and integrate both genetic and environmental factors to clarify the mechanisms underlying these associations.

**Systematic review registration:**

https://www.crd.york.ac.uk/prospero/, identifier CRD420251020891.

## 1 Introduction

### 1.1 The metacognitive model of psychopathology

Metacognition is the set of frameworks, content, and procedures that support the monitoring, evaluation, and regulation of cognition (1, 2). The metacognitive model suggests that metacognitions play a central role in sustaining psychological distress and influencing biased cognitive processing (3, 4). In order to explain how metacognitive processes contribute to the maintenance and the control of emotional disorders, Wells and Matthews (4, 5) elaborated the Self-Regulatory Executive Function model (S-REF). The S-REF model emphasizes that maladaptive top-down control over attention and cognition, rather than automatic bottom-up biases, lies at the core of many psychological difficulties. In its most recent elaboration, Wells further develops the S-REF model by clarifying its functional architecture, specifically through the interaction between the Metacognitive Control System (MCS) and the Cognitive System (CS) (2). The CS comprises both low-level automatic processes and online strategic processing, which occur within a limited-capacity “thinking space.” The MCS continuously monitors CS activity and directs it toward the attainment of self-regulatory goals, drawing on metacognitive knowledge stored in long-term memory. Its primary function is to detect and respond to mismatches between desired goals and the current state (2). When such a mismatch is identified, it is signaled to the CS through an additional component of the MCS, referred to as the cybernetic code. This code can activate commands that bias attention toward particular internal or external stimuli—such as bodily sensations or specific thoughts—in order to resolve discrepancies or sustain goal-directed processing. By biasing attention, the system can maintain or adjust processing routines, for instance by focusing on threat-related cues or internal signals of conflict, thereby supporting self-regulation (2). The action of the cybernetic code is repeated in a loop through a continuous feedback process managed by the MCS, and under normal, non-pathological conditions, this cycle naturally comes to an end once the self-regulatory goal is achieved (2).

However, the system fails to achieve effective self-regulation when attention becomes excessively rigid due to the influence of dysfunctional metacognitive knowledge. In such cases, dysfunctional metacognitive beliefs lead self-regulation strategies to be dominated by the Cognitive Attentional Syndrome (CAS; 6). CAS represents a transdiagnostic thinking style characterized by perseverative thinking (e.g., worry, rumination), self-focused attention, thought suppression, reassurance seeking, and avoidance.

According to Wells and Cartwright-Hatton (7), dysfunctional metacognitive beliefs broadly take five different forms: positive beliefs about the usefulness of engaging in worry and/or rumination (POS), negative beliefs about uncontrollability and danger of thoughts (NEG), need to control thoughts (NC), low cognitive confidence (CC), and cognitive self-consciousness (CSC). POS involve beliefs that worry or rumination are beneficial (e.g., “Worrying helps me stay in control”; “Rumination can help me to find a solution”), often framing them as useful problem-solving tools (7). NEG, on the other hand, reflect beliefs about the harmful and uncontrollable nature of worry and/or rumination (e.g., “Worrying will drive me crazy”, “If I continue to ruminate I will lose my mind”) (4, 5, 7). The NC refers to the belief that unwanted thoughts are dangerous, unacceptable, or harmful, and therefore must be eliminated or strictly controlled (e.g., I should be in control of my thoughts all of the time) (4, 5, 7). CC, especially in relation to memory and concentration (e.g., “My memory can mislead me at times”), contributes to increased repetitive negative thinking and efforts to regulate thoughts (1, 4, 5). CSC, or heightened awareness and monitoring of one’s thoughts (e.g., “I pay close attention to the way my mind works”), is associated with pathological worry and further promotes repetitive thinking patterns (3–5).

When such metacognitive beliefs are activated, the CAS becomes the dominant self-regulatory strategy, yet this process is ineffective as it exacerbates the perceived discrepancy from the desired state, thereby rendering the cybernetic looping a self-perpetuating cycle in which the individual becomes entrapped (2).

Among the dysfunctional metacognitive beliefs, POS and NEG are considered the most clinically relevant, as they not only sustain worry and rumination but also drive the development of the other metacognitive mechanisms (2, 7). Coherently with the S-REF model (4, 5), metacognition has initially been investigated as a transdiagnostic feature of emotional disorders, such as generalized anxiety disorder (GAD), obsessive-compulsive disorder (OCD), and depression (8). Subsequent research has further explored the association between metacognition and a broader spectrum of psychological disorders. In particular, dysfunctional metacognitive beliefs have been identified as a key maintenance factor in eating disorders (9–11) and are closely linked to difficulties in emotional regulation (12–14). Moreover, maladaptive beliefs about one’s own cognitive and emotional states appear to play a crucial role also in behavioral problems such as aggression (15, 16) and addictive behaviors (17–19).

### 1.2 The metacognitive model in childhood and adolescence

Despite the recognition of the influence of dysfunctional metacognitive beliefs on a wide range of psychopathological conditions, which are therefore highly heterogeneous, current knowledge about their interaction with psychopathological trajectories remains limited. This is largely due to the fact that, until recently, research has primarily focused on adult populations. Only in more recent years has the metacognitive model (4, 5) been extended to younger age groups, with studies evaluating its applicability to children and adolescents. However, these studies have concentrated almost exclusively on emotional symptoms (20–22). The main results of these works highlight that children as young as 7 years of age have the ability to formulate both positive and negative beliefs about their thoughts and their worry (7), and these beliefs would be associated with an increase in anxiety symptoms (22, 23). Moreover, additional research suggests that clinically anxious youths report higher levels of metacognitive beliefs than nonanxious youths, regardless of anxiety disorder. For example, Esbjørn et al. (21) sampled 69 Danish children aged 7 to 12 and found that children with GAD have significantly higher levels of deleterious metacognitions than anxious children without a diagnosis and nonanxious children. More recently, the same research group (24) found that social anxiety symptoms correlated positively with social threat, negative automatic thoughts and negative metacognitive beliefs, and negatively with positive automatic thoughts in a sample of 122 children aged 7–13 years. The relationship between dysfunctional metacognitive beliefs and behavioral problems in this age group is still poorly understood. However, emerging evidence suggests a positive association during adolescence as well (25), with some studies also supporting the effectiveness of metacognitive therapy in treating these symptoms (26).

Further evidence of the contribution of metacognitive beliefs to anxiety and depressive symptoms from childhood through adolescence is provided by a recent meta-analysis by Thingbak et al. (27) This study confirms that the existing literature highlights a significant association between dysfunctional metacognitive beliefs and symptoms of anxiety and depression in populations aged 7–18 years. In addition, the authors report that levels across various domains of dysfunctional metacognitive beliefs tend to be higher among children and adolescents in clinical populations diagnosed with anxiety or depression, compared with their counterparts in the general population.

### 1.3 Current insights into the origins of metacognitive beliefs

As just reported, progress has been made in understanding the involvement of metacognitive patterns in the etiology of psychopathological traits in childhood and adolescence. However, it remains unclear how metacognitive beliefs emerge and become problematic during development, although the processes through which this involvement is expressed are being increasingly delineated (2). What still requires clarification are the factors that influence the development of metacognitive beliefs from early childhood onward. At present, most studies addressing this issue seem to rely on the hypothesis that the origin of metacognitive beliefs lies in exposure to familial environmental factors that predispose individuals to the acquisition of such dysfunctional thought patterns, drawing on the literature concerning the transmission of cognitive biases (28, 29).

These studies suggest that one of the main factors underlying this mechanism is children’s direct verbal exposure to their parents’ cognitive distortions, through which they internalize threatening messages and consequently shape their own interpretive biases (30–32).

At present, only limited evidence supports a direct association between parenting styles characterized by excessive criticism or overinvolvement and children’s metacognitive beliefs. The proposed interpretation is that such parental behaviors may discourage the use of active coping strategies, thereby fostering the development of dysfunctional metacognitive beliefs (33). These beliefs, in turn, sustain an internalized mode of managing emotional distress, as they lead children to perceive themselves as less capable of autonomously and effectively coping with negative emotional states (33). Overall, however, findings from studies that have directly investigated the association between parents’ and children’s metacognitive beliefs are mixed, preventing firm conclusions about the nature of this relationship and the mechanisms responsible for it.

Another limitation of the existing literature on the origins of metacognitive beliefs, as can be observed, is that the role of genetics is often overlooked. Although environmental influences are undoubtedly significant, it is well established that cognitive abilities arise from the interaction between genetic and environmental factors, an interplay that is particularly influential in shaping the cognitive profile of offspring from childhood through adolescence (34–36).

The genetic contribution to thought processes associated with cognitive distortions underlying psychopathology has been widely demonstrated by various twin studies. These studies estimate genetic and environmental influences on phenotypes by comparing monozygotic twins, who originate from a single zygote, with dizygotic twins, who develop from two different zygotes. Findings consistently show that the components of CAS have a substantial heritable component, with estimates ranging from 20% to over 40% of the total variance explained by genetic factors (37–39).

Nevertheless, to date, studies that have directly investigated the association between genetic factors and the components of the metacognitive model remain markedly insufficient. However, as described in the metacognitive model, metacognitive beliefs are closely connected to cognitive abilities and executive control, particularly to attentional processes, whose interaction is thought to underlie the regulation of the cybernetic loop (2). This framework, in turn, makes it plausible to hypothesize an involvement of genetic components related to attentional processes in shaping metacognitive beliefs. Preliminary evidence suggests that two specific polymorphisms of the DRD4 gene—namely, the 2-repeat and 7-repeat variants—are associated with lower levels of metacognitive beliefs (40), as measured by the Metacognitions Questionnaire (MCQ-30,41). The literature indicates that these particular variants of the DRD4 gene are linked to reduced dopamine receptor function, which in turn is associated with increased impulsivity, novelty-seeking behavior, and less effective attentional control (41–43).

### 1.4 Objective and hypotheses

In light of the above, this systematic review and meta-analysis aims to provide a comprehensive overview of the studies conducted to date on the intergenerational dimension of metacognitive beliefs, thereby laying the groundwork for future research to enrich the metacognitive framework by incorporating crucial insights into their origins.

More specifically, we examined studies that focused on the association of metacognitive beliefs, as described in the S-REF model (2, 4, 5), either between parents and children or between these beliefs and genotype, with the aim of providing a precise estimate of the overall effect size reflecting the strength of the association. Additionally, we sought to determine whether these estimates were influenced by the presence of an emotional disorder within the sample, given that such diagnoses have been shown to significantly influence levels of dysfunctional metacognitive beliefs, as reported in the literature (27).

Finally, we examined the moderating effects of two primary variables. First, age: although prior studies report no significant impact on the association between metacognitive beliefs and anxiety/depressive symptoms, indicating relative temporal stability of the S-REF framework (44), behavioral genetics suggests that familial environmental influences on genotypes diminish over time (45). Second, the risk of bias in individual studies, which has been shown to affect overall effect sizes (46). Given the subjective nature of this index, which warrants caution in its use (47), we further examined a related factor, namely the number of covariates included in each study, to assess its potential influence on the effect estimates.

We hypothesized that: 1) a significant association would be observed across all dimensions of metacognitive beliefs, both between parents and children and in relation to participants’ genotype, in line with the literature on the intergenerational link of cognitive distortions and twin studies on the components of the CAS; 2) this association would be more pronounced in clinical populations, as it is plausible to assume that individuals with a diagnosis are exposed to both greater genetic vulnerability and an environment likely to facilitate the acquisition of dysfunctional metacognitive beliefs.

## 2 Materials and methods

### 2.1 Identification and selection of studies

Study selection methodology followed the PRISMA guidelines (48).

#### 2.1.1 Eligibility criteria

To guide the study selection process, the following eligibility criteria were established in line with the study objectives: 1) the studies had to contain an effect size (ES) related to the intergenerational dimension, assessed through the relationship either between metacognitive beliefs and genotype or between parents’ and children’s metacognitive beliefs. If the genotype was not involved, the studies had to include an assessment of metacognitive beliefs for both parents and children; 2) the sample had to include individuals aged 7 years and older (no upper limit was imposed), as this is the age at which they begin to develop the ability to formulate both positive and negative beliefs about their thoughts and worries (20, 22, 23); 3) the included studies could involve both general and clinical populations, as dysfunctional metacognitive beliefs are not necessarily limited to clinical populations but can also be present in the general population; 4) all the articles had to be published in peer-reviewed international journals; 5) in defining metacognitive beliefs the authors had to refer to the S-REF model (4, 5); 6) the articles had to be written in English.

The following exclusion criteria were applied: 1) studies not reporting a direct estimate of the intergenerational association of metacognitive beliefs or not including an assessment measure of children’s metacognitive beliefs and those of at least one parent; 2) studies not clearly referencing the S-REF model (4, 5) in defining the metacognition construct; 3) studies that were systematic reviews, theses, dissertations, or meta-analyses; 4) studies that were not written in English.

#### 2.1.2 Information sources and search strategies

The studies were identified through a comprehensive literature search conducted using the following electronic databases: PubMed, EBSCOhost (including the databases APA PsycInfo, APA PsycArticles, PSYNDEX Literature with PSYNDEX Tests, and ERIC), and SCOPUS. The last search was conducted on April 24^th^ 2025. Additionally, we reviewed the references to identify further relevant literature.

To conduct the search, the following keywords were used: (“metacognitive beliefs” OR metacognition OR “metacognitive processes” OR “cognitive monitoring” OR “metacognitive regulation” OR “positive beliefs about worry” OR “negative beliefs about worry”) AND (transmission OR “parent-to-child transmission” OR “family influence” OR “generational influence” OR “transgenerational patterns”).

To ensure that as much literature as possible was considered, no filters were applied regarding participants’ age, population characteristics (clinical or general population), or study design. However, in line with the inclusion criteria, filters were applied to restrict the search to studies conducted on humans, published in academic peer-reviewed journals, and written in English.

Additionally, only in SCOPUS, which was the only database offering this option, filters were applied to limit the search to articles related to psychology, medicine, or neuroscience. In all databases, a filter was applied to restrict the search to articles published from 1994 onward, the year in which Wells & Matthews introduced the S-REF model (4). The systematic review protocol was registered in the International Prospective Register of Systematic Reviews (PROSPERO) under the registration number CRD420251020891.

#### 2.1.3 Selection process

The article screening procedure was conducted independently by two researchers. After the removal of duplicates, articles were screened based on their titles and abstracts. Finally, a full-text assessment was conducted on the remaining studies, and suitable articles were selected and included in the systematic review.

All disagreements on eligibility were resolved by consensus.

#### 2.1.4 Data item

Any measure of the degree of association between metacognitive beliefs and genotype, or between parental and offspring metacognitive beliefs, was considered eligible for inclusion in the review. No restrictions were applied regarding the method used to assess metacognitive beliefs, except that the instruments had to explicitly refer to the construct of metacognition as theorized within the S-REF model (4, 5). No limitations were imposed concerning the specific dimension of metacognitive beliefs investigated in the individual studies; thus, studies that examined only some domains of metacognitive beliefs or metacognitive beliefs as a whole were included. The only restriction concerned the timing of the measurement of metacognitive beliefs in the offspring, which had to start from the age of 7 years and could occur at any phase of the study (baseline or follow-up).

### 2.2 Risk of bias assessment

Two independent researchers assessed the quality of the individual included studies using the Newcastle-Ottawa Scale (NOS; 49). The NOS evaluates three quality parameters—selection, comparability, and outcome—distributed across multiple items. Each item is awarded one point, except for comparability and ascertainment of exposure (risk factor), which can be adjusted based on the research topic and assigned up to two points. The maximum possible score is 10 points for cross-sectional studies and 8 for longitudinal studies, with those scoring less than 5 points classified as being at high risk of bias.

### 2.3 Effect measures and data analyses

The Pearson correlation coefficient (r) or the standardized regression coefficient (β) were selected as effect size measures. A narrative synthesis of all included studies was therefore conducted, and, where the quantitative data could be validly combined, they were analyzed using meta-analytic techniques. More precisely, this type of analysis was conducted using the “metafor” (50) and “robumeta” (51) packages for R (52). The linear relationship coefficients from each study were converted into Fisher’s z, and then the overall Fisher’s z was calculated. Afterwards, this value was converted back into general correlation values. The overall effect sizes were evaluated based on Cohen’s criteria (53), according to which an effect size (ES) between.10 and.30 is considered small, an ES between.30 and.50 is considered medium, and an ES greater than.50 is considered large.

Random-effects model was applied for the analyses, as the data were obtained from studies involving different populations. The Q statistic was used to test the heterogeneity of the effect sizes as well as the effect of selected *a priori* moderators. To obtain a more accurate measure of the heterogeneity of the included studies, the I² index was also calculated. This index measures the proportion of total variance attributable to real differences between studies rather than within-study variance (54, 55). Unlike the Q test, the I² index is not affected by the number of included studies, provides an estimate of the percentage of variance, and allows for the calculation of confidence intervals (CIs) (55).

Following the guidelines of Higgins et al. (56), an I² value of 25%, 50%, and 75% represents a low, moderate, and high degree of variance between studies, respectively. In the case of significant heterogeneity among studies, the effect of the previously described moderators was evaluated.

To assess the presence of potential publication bias (the phenomenon in which studies with stronger effect sizes are more likely to be published and therefore included in the meta-analysis) a visual analysis of the funnel plot was first conducted. Generally, if the distribution of effect sizes within the plot is symmetrical, this indicates the absence of publication bias.

The asymmetry of the funnel plot was tested using Egger’s regression test (57), which, compared to other inferential tests, is the most suitable for meta-analyses with a small number of included studies (55).

In cases where publication bias was detected, the trim-and-fill procedure (58) was applied. This method replaces extreme studies in the funnel plot with imputed studies to increase symmetry, allowing for the computation of an adjusted effect size (ES) and its corresponding CI (54, 55).

The results were combined using the types of metacognitive beliefs (i.e., POS, NEG, CC, NC, CSC) as the grouping criterion. The results from the meta-analytic analyses were presented in a dedicated section of the paper.

## 3 Results

### 3.1 Study selection

The literature search initially yielded 1,084 articles from PubMed (n = 15), EBSCOhost (n = 153), and SCOPUS (n = 916) databases, resulting in 1,067 articles after duplicate removal. Of these, 1,017 were excluded based on the title and abstract. The remaining fifty articles underwent full-text screening. Among these, seven articles met all inclusion criteria and were included in the systematic review.

Of the fifty articles initially identified as eligible, thirty-two were excluded because they did not refer to the S-REF model (4, 5), one was excluded because it did not include a measure of children’s metacognitive beliefs, two were excluded due to the absence of a measure of parental metacognitive beliefs, six were excluded as they were literature reviews, and two were excluded because, even after contacting the authors, it was not possible to obtain the full text of the paper.

The reference lists of the included articles were also screened, and through citation searching, four additional potentially eligible records were identified. Of these four, one was excluded based on the abstract. The remaining three articles underwent full-text screening, and one of them was excluded because it did not report outcome data on the intergenerational dimension of metacognitive beliefs. In total, nine articles were ultimately included in this systematic review (28, 29, 33, 59–64).

**Figure 1:**
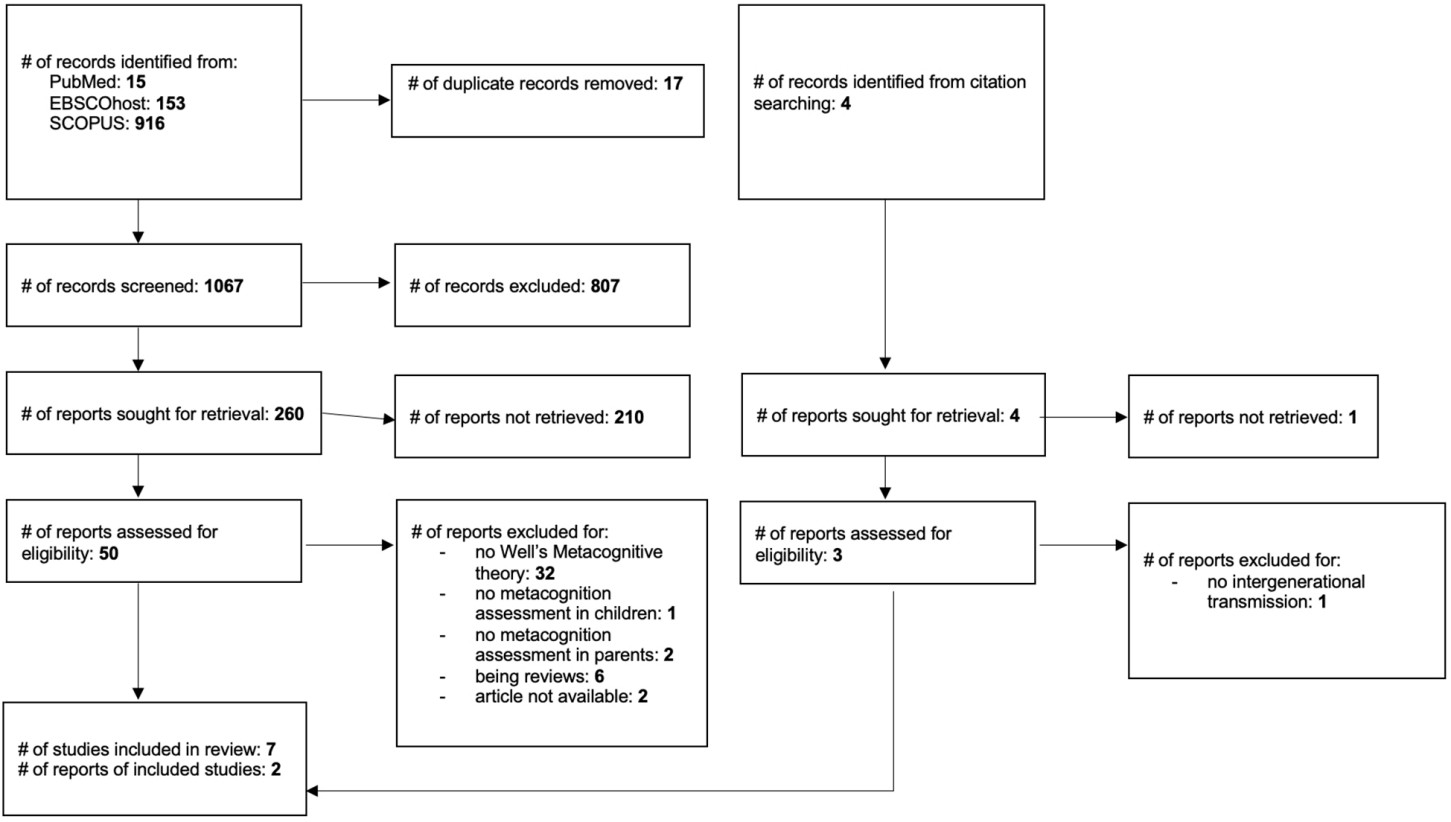
PRISMA flowchart: study selection.

The PRISMA flowchart showing the selection process is presented in **Figure 1**.

### 3.2 Studies characteristics

Among the included studies, eight had a cross-sectional design (28, 33, 59–64), while one had a longitudinal design (29). All studies were published between 2006 and 2023, well after the publication of the S-REF model (4, 5). The origin of the studies is quite heterogeneous: four studies come from Europe—specifically, three from Denmark (28, 29, 64) and one from Germany (62). Two of the three Danish studies (28, 29) used samples drawn from the same general population cohort, while, Lønfeldt et al. (64) drew on an expanded sample that included all participants from the study by Esbjørn et al. (28). The remaining five studies originated from the United Kingdom (63), the USA (60), Hong Kong (33), Lebanon (61), and Australia (59).

Eight studies assessed the association between parents’ metacognitive beliefs and those of their children (28, 29, 33, 59, 60, 62–64), while only one investigated the association between metacognitive beliefs and participants’ genotype (61).

Seven out of nine studies involved parent-child dyads from the general population (28, 29, 33, 59, 60, 63, 64), one used a mixed sample (62), and one used exclusively clinical samples (61). Specifically, Köcher et al. (62) compared a sample of children and adolescents diagnosed with an anxiety disorder to a non-clinical sample, while the study by Fekih-Romdhane et al. (61) focused on a clinical sample of individuals with schizophrenia.

Regarding the assessment of metacognitive beliefs, almost all included studies (28, 59, 61–64) used the Metacognition Questionnaire (MCQ-30; 41), in both adult and child/adolescent versions. Jacobi et al. (60) administered the Cognitive Self-Consciousness Scale-Expanded (CSC-E), a scale derived from the CSC subscale of the MCQ (65), with the addition of seven items from the Pain Vigilance and Awareness Questionnaire (66, 67). In the study by Chow and Lo (33), participants were administered the Positive & Negative Beliefs about Rumination Scale (PBRS & NBRS; 68,69), a tool more specifically tailored to assess positive and negative thoughts about rumination.

Three out of nine studies (28, 29, 64) included dyads composed exclusively of children and their mothers, whereas in five studies (33, 59, 60, 62, 63) dyads could also involve fathers, although 80–90% still consisted of mothers.

Overall, the included studies analyzed 911 parent-child dyads. The age range of the children was from 8 to 16 years; not all studies reported the age of the parents involved, but based on available data, it ranged approximately from 28 to 63 years. The 115 patients in the study by Fekih-Romdhane et al. (61) were all adults with a mean age over 50 years.

Regarding outcome measures, eight out of nine studies (28, 29, 33, 59, 60, 62–64) measured the intergenerational dimension of metacognitive beliefs by estimating Pearson’s *r* between children’s and parents’ metacognitions. The study by Fekih-Romdhane et al. (61), on the other hand, reported the standardized regression coefficient (β) between metacognitive beliefs and a specific polymorphism of the Catechol-O-Methyltransferase (COMT) gene rs4680.

### 3.3 Risk of bias in individual studies

None of the studies fulfilled all the Newcastle-Ottawa quality criteria, reaching the maximum possible score. However, none scored below 5, meaning no included study can be considered at high risk of bias. Of the eight cross-sectional studies, two scored 5/10, two scored 6/10, two scored 7/10, and two scored 8/10. The only included longitudinal study scored 5/8. Further details regarding study quality for the selected studies can be found in **Table 1**.

**Table 1:** Quality assessment using the Newcastle-Ottawa scale.

Cross-sectional studies										
Study	Selection				Comparability	Outcome				
Author(s), year	Representativeness of the sample	Sample size	Non-respondents	Ascertainment of the exposure (risk factor)	Study design and analyses	Assessment of the outcome		Statistical test		Total score
Jacobi et al., 2006 (60)	*			**		*		*		5/10
Wilson et al., 2011 (63)	*			**	*	*		*		6/10
Esbjørn et al., 2016 (28)	*			**	*	*		*		6/10
Donovan et al., 2017 (59)	*		*	**	**	*		*		8/10
Chow and Lo., 2017 (33)	*			**		*		*		5/10
Lønfeldt et al., 2017 (64)	*			**	**	*		*		7/10
Köcher et al., 2023 (62)	*		*	**	**	*		*		8/10
Fekih-Romdhane et al., 2024 (61)	*			**	**	*		*		7/10
Longitudinal studies										
	Selection				Comparability	Outcome				
Study	Representativeness of the sample	Selection of the non exposed cohort	Ascertainment of exposure	Demonstration that outcome of interest was not present at start of study	Study design and analyses	Assessment of outcome	Time before Follow-up		Adequacy of follow up of cohorts	Total Score
Walczak et al., 2021 (29)	*	Non-applicable		Non-applicable	**		*		*	5/8

### 3.4 Results of individual studies

The characteristics and the results of the individual studies included are presented in more detail in **Table 2**. In the following sections of the paper, a summary of the results will be presented in narrative form, along with the results of the meta-analysis for the subgroup of the articles that could be analyzed (see section 3.5).

**Table 2:** Characteristics of the nine studies included in the systematic review.

Study	ID	Nation	Design	Sample	Gender_c_	Mean Age_c_ (SD) [Range]	Gender _p_	Mean Age_p_ (SD) [Range]	Measures	Outcome	Results
Chow and Lo., 2017 (33)	1	Hong Kong	cross-sectional	85 child-parent dyads	female n = 50 male n = 32	13.2 (1.2) [11 – 16]	female n = 63 male n = 22	45.0 (5.67) [NR]	PBRS, NBRS	Pearson’s *r* correlation coefficient	PBRp-PBRC: r=.33, p<.01; NBWp-NBWc: r=.23, p<.05
Donovan et al., 2017 (59)	2	Australia	cross-sectional	114 child-parent dyads	female n = 58 male n = 56	9.87 (1.30) [8 – 12]	female n = 102 male n = 11 unidentified = 1	40.96 (4.84) [28 – 56]	MCQ-C, MCQ-30	Pearson’s *r* correlation coefficient	PBWp-PBWc: r= -.12, p>.05; NBWp-NBWc: r=.13, p>.05
Esbjørn et al., 2016* (28)	3	Denmark	cross-sectional	111 child-mother dyads	female n = 71 male n = 40	10.05 (1.42) [NR]	female n = 111	42.45 (4.51) [NR]	MCQ-C_30_, MCQ-30	Pearson’s *r* correlation coefficient	M-TOTm-M-TOTc: r=.38, p<.01; PBWm-PBWc: r=.27, p<.01; NBWm-NBWc: r=.22, p<.05; CCm-CCc: r=.30, p<.01; CSCm-CSCc: r=.30, p<.01; NCm-NCc: r=.15, p>.05
Fekih-Romdhane et al., 2024* (61)	4	Lebanon	cross-sectional	115 patients with schizofrenia	female n = 42 male n = 73	57.64 (10.41) [NR]	–	–	MCQ-30	Standardized β regression coefficient	CC-AG*COMT rs4680 gene: β = 7.57, p <.05 NC-AG*COMT rs4680 gene: β = 9.87, p <.05
Jacobi et al., 2006 (60)	5	USA	cross-sectional	126 child-parent dyads	female n = 87 male n = 39	16.2 (1.2) [NR]	female n = 105 male n = 21	45.7 (4.3) [NR]	CSC-E	Pearson’s *r* correlation coefficient	CSC-E_p_-CSC-E_c_; r=.06, p>.05
Köcher et al., 2023 (62)	6	Germany	cross-sectional	clinical: 68 child-parent dyads	female n = 42 male n = 26	11.89 (2.43) [8 – 16]	female n = 60 male n = 8	41.57 (5.25) [31 – 51]	MKF-K, MKF-30	Pearson’s *r* correlation coefficient	Clinical Sample: PBWp-PBWc: r=.18, p>.05; NBWp-NBWc: r=.24, p<.05 Non Clinical Sample: PBWp-PBWc: r=.54, p<.01; NBWp-NBWc: r=.38, p<.01
				non clinical: 40 child-parent dyads	female n = 19 male n = 21	11.54 (1.54) [9 –15]	female n = 39 male n = 1	43.05 (6.86) [30 – 63]			
Lønfeldt et al., 2017 (64)	7	Denmark	cross-sectional	188 child-mother dyads	female n = 104 male n = 84	10.01 (1.41) [7 – 12]	female n = 188	no data	MCQ-C_30_, MCQ-30	Pearson’s *r* correlation coefficient	PBWm-PBWc: r=.29, p <.01; NBWm-NBWc: r=.16, p <.05; CCm-CCc: r=.22, p <.01; CSCm-CSCc: r=.24, p <.01; NCm-NCc: r=.14, p>.05
Walczak et al., 2021* (29)	8	Denmark	longitudinal	107 child-mother dyads	female n = 65 male n = 42	at follow-up 13.1 (1.4) [NR]	female n = 107	no data	MCQ-C_30_, MCQ-30	Pearson’s *r* correlation coefficient	Baseline M-TOTm-M-TOTc: r=.27, p<.01 Follow-Up M-TOTm-M-TOTc: r=.26, p<.01
Wilson et al., 2011 (63)	9	United Kingdom	cross-sectional	72 child-parent dyads	female n = 39 male n = 33	13.2 (1.04) [11 – 16]	female n = 62 male n = 10	no data	MCQ-A, MCQ-30	Pearson’s *r* correlation coefficient	PBWp-PBWc: r=.27, p>.05; NBWp-NBWc: r=.03, p>.05; CCp-CCc: r=.04, p>.05; CSCp-CSCc: r=-.09, p>.05; NCp-NCc: r=.13, p>.05

NR: not reported; _c_:children; _p_: parents; _m_: mothers; CSC-E: Cognitive Self-Consciousness Scale-Expanded (65); MCQ-30: Meta-Cognitions Questionnaire-30 item version (7); MCQ-A: Meta-cognitions questionnaire-adolescent version (68); MCQ-C: Meta-Cognitions Questionnaire for Children (69); MCQ-C_30_: Metacognitions Questionnaire for Children – 30 item version (70); MKF-30: German Short Form of the Metacognitions Questionnaire (71); MKF-K: German Metacognitions Questionnaire for children (72); NBRS: Negative Beliefs about Rumination Scale (73); PBRS: Positive Beliefs about Rumination Scale (74).

*Studies not analyzed through the meta-analytic method due to heterogeneity in design and outcome measures, and to sample overlap.

### 3.5 Narrative synthesis of results

Not all of the included studies assessed the same dimensions of metacognitive beliefs. Some examined the intergenerational association across all subscales of the MCQ-30, whereas others relied solely on the total scale score. In contrast, certain studies focused exclusively on either POS or NEG.

#### 3.5.1 Overall metacognitive beliefs

Of the nine studies included, only two also tested the association between parents’ and children’s metacognitive beliefs by considering metacognition as a unitary construct. Specifically, Esbjørn et al. (28), in addition to analyzing individual domains, also examined the association between the total MCQ-30 scores of mothers and their children, finding a significant correlation coefficient of.38 (p <.01). These findings were corroborated by Walczack et al. (29), who used the same sample and relied solely on the total MCQ-30 score. They reported a significant association between mothers’ and children’s overall metacognitive beliefs, although with a smaller magnitude than the previous study (r = .27; p <.01), which remained stable over a three-year follow-up period (r = .26; p <.01).

#### 3.5.2 Positive beliefs about the usefulness of engaging in worry and rumination

An estimate of the intergenerational association of POS was reported in seven out of the nine studies. Both Esbjørn et al. (28) and Lønfeldt et al. (64) found significant positive associations between mothers and children in this domain of metacognitive beliefs, with reported correlations of r = .27 (p <.01) and r = .29 (p <.01), respectively. Chow and Lo (33) also observed a significant association between parents’ and children’s positive metacognitive beliefs related to rumination (r = .33, p <.01).

Köcher et al. (62) examined the relationship between parents’ and children’s metacognitive beliefs in both clinical and non-clinical samples. They found a strong and statistically significant association in the non-clinical sample (r = .54, p <.001), whereas the association did not reach statistical significance in the clinical sample (r = .18).

Wilson et al. (63) did not find a statistically significant association, although the observed correlation (r = .27) was the highest among all metacognitive belief domains assessed in that study. Also Donovan et al. (59) reported a negative, non-significant correlation between parents’ and children’s positive metacognitive beliefs (r = –.12). Lastly, Fekih-Romdhane et al. (61) found no significant association with the AG polymorphism of the COMT rs4680 gene (β = 5.84, p = .055).

#### 3.5.3 Negative beliefs about uncontrollability and danger of thoughts

The intergenerational association concerning NEG was examined in seven of the nine studies included in the review.

Again, both Esbjørn et al. (28) and Lønfeldt et al. (64) reported significant positive associations between mothers and children, with correlations of r = .22 (p <.05) and r = .16 (p <.05), respectively. Chow and Lo (33), as well, found a significant association between parents’ and children’s negative metacognitive beliefs about rumination (r = .23, p = .03).

Köcher et al. (62) observed results similar to those for POS: the association was stronger in the non-clinical sample (r = .38, p <.01), while in the clinical sample the association was also significant, albeit weaker (r = .24, p <.05).

Wilson et al. (63) and Donovan et al. (59) both reported non-significant correlations, with very small effect sizes (r = .03 and r = .13, respectively).

Lastly, Fekih-Romdhane et al. (61) once again found no significant association with the AG polymorphism of the COMT rs4680 gene (β = 4.97, p = .121).

#### 3.5.4 Cognitive confidence

The intergenerational dimension of CC was explored in four of the nine included studies.

Like in the previous cases, both Esbjørn et al. (28) and Lønfeldt et al. (64) observed significant positive associations between mothers and children, with Pearson’s correlation coefficients of.30 (p <.01) and.22 (p <.01) respectively. In addition, CC was found to be significantly associated with the AG polymorphism of the COMT rs4680 gene, with Fekih-Romdhane et al. (61) reporting a significant effect (β = 7.57, p = .011), suggesting a potential genetic contribution to this specific domain of metacognitive beliefs.

Only Wilson et al. (63) found no significant association, with a very small correlation (r = .04).

#### 3.5.5 Need to control thoughts

NC was assessed in four of the nine studies reviewed.

The only study to report a significant association was the one by Fekih-Romdhane et al. (61), who found that this specific metacognitive domain was significantly associated with the AG polymorphism of the COMT rs4680 gene (β = 9.87, p = .030). In contrast, Wilson et al. (63), Esbjørn et al. (28) and Lønfeldt et al. (64) found no significant associations between parents and children, reporting correlation coefficients of.13,.15, and.14, respectively.

#### 3.5.6 Cognitive self-consciousness

Evidence regarding the intergenerational association of CSC was available in five of the nine studies reviewed.

Esbjørn et al. (28) identified a significant positive correlation (r = .30, p<0.01), as well as Lønfeldt et al. (64) that reported a significant positive association between mothers and children (r = .24, p <.01), representing the strongest intergenerational link found among all metacognitive domains assessed in their study.

Jacobi et al. (60), which was the only study among the included ones that focused exclusively on this specific type of metacognitive beliefs found no significant association (r = .06). Similarly, Wilson et al. (63) reported a non-significant correlation (r = –.09), jus as in the previous cases.

In terms of genetic associations, Fekih-Romdhane et al. (61) once again found no significant link with the AG polymorphism of the COMT rs4680 gene (β = –.68, p = .895).

### 3.6 Meta analysis results

Three studies discussed narratively were not included in the meta-analysis, primarily due to heterogeneity in design and outcome measures, and to sample overlap. For the former reason, we excluded Fekih-Romdhane et al. (61), the only study investigating the association between metacognitive beliefs and genotype. Walczak et al. (29) was also excluded, as it assessed metacognitive beliefs exclusively through the MCQ-30 total score and employed a longitudinal design with a baseline sample overlapping with Esbjørn et al. (28), which was in turn excluded because its sample was later incorporated into Lonfeld et al. (64), the study retained for analysis following the guidelines by (47).

In line with recommendations warning that meta-analyses based on very few studies provide insufficient power to estimate heterogeneity and carry a high risk of misleading conclusions (75, 76), we limited the analyses to domains represented by at least three studies. Accordingly, meta-analyses were conducted for the POS, NEG, and CSC domains.

Finally, given that only one clinical sample was available (from the study by Köcher et al. (62)), it was not possible to conduct a subgroup analysis to explore differences in effect sizes between clinical and general populations.

#### 3.6.1 Positive beliefs about the usefulness of engaging in worry and rumination

The meta-analysis for this metacognitive domain included six samples from five studies (33, 59, 62–64), including both the clinical and general population samples from Köcher et al. (62)

**Figure 2:**
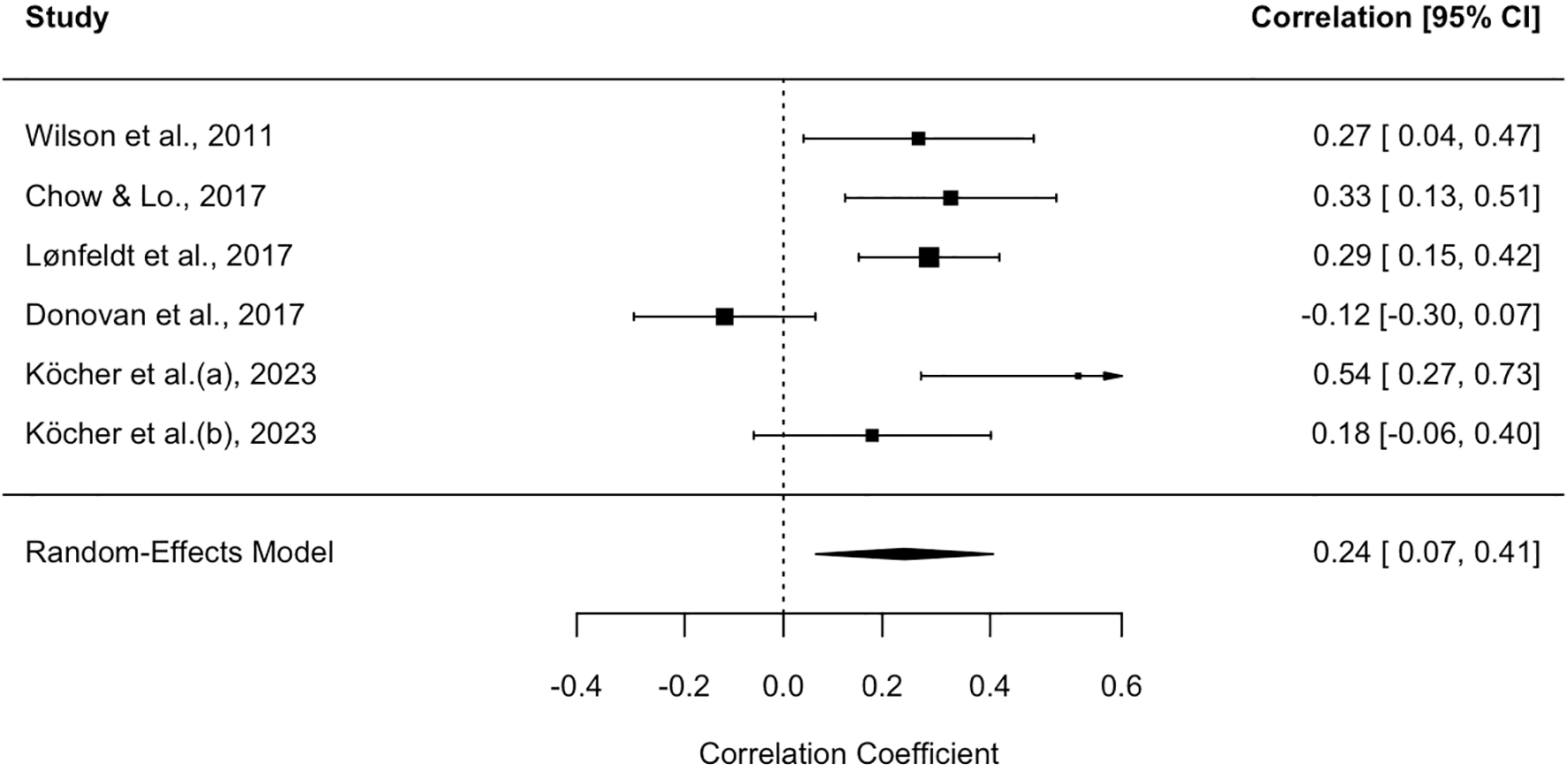
Forest plot for the studies on positive beliefs about the usefulness of engaging in worry and rumination. Each study included in the meta-analysis is represented by a point estimate, which is bounded by a 95% CI. The summary effect size is displayed as a polygon at the bottom of the plot, with the width of the polygon representing the 95% CI.

As reported in **Figure 2**, the relationship between parents’ and children’s positive metacognitive beliefs reported an average effect size of.24 (SE = .09, 95% CI = .06,.43; test of null (2-tail): z-value = 2.68, p <.01, k = 6), indicating a small but relevant connection between parents’ and children’s POS.

Due to the significant heterogeneity observed among the studies (I² = 76.9%, CI: 38.78, 96.49; Q = 21.27, p <.01), further analyses were conducted to explore potential moderators. Specifically, meta-regressions performed on the continuous variables “children’s age” (β = .05, SE = .06, 95% CI = -.06,.20, z-value = .99, p = .32) and “quality score of the studies” (β = -.50, SE = .85, 95% CI = -2.17, 1.17, z-value = -.58, p = .55) were not statistically significant.

Similarly, the meta-regression on the categorical variable “number of covariates” (β_1_ [multiple covariates] = -.11, SE = .32, 95% CI = -.74,.51, z-value = -.37, p = .71; β_2_ [one covariate] = -.06, SE = .40, 95% CI = -.86,.73, z-value = -.16, p = .87) also yielded non-significant results. These findings suggest that the analyzed factors may not be the primary drivers of the observed variability, or alternatively that the statistical power of the meta-regression was insufficient to detect potential moderating effects.

To address a potential publication bias, the funnel plot (**Supplementary Figure S1**, in **Supplementary Material**) was examined, revealing a slight asymmetry between the studies. Although Egger’s test was not significant (b = –.22, 95% CI = –.97,.54, z = 1.24, p = .21), since the visual inspection of the plot could raise some concerns, given the small number of studies included the trim-and-fill procedure was also applied, which suggested the possible presence of missing studies.

After correction, the adjusted effect size (adjES = .16, 95% CI = –.03,.34) was found to be non-significant (p = .09), suggesting the possibility that publication bias may have influenced the original effect size estimate.

#### 3.6.2 Negative beliefs about uncontrollability and danger of thoughts

**Figure 3:**
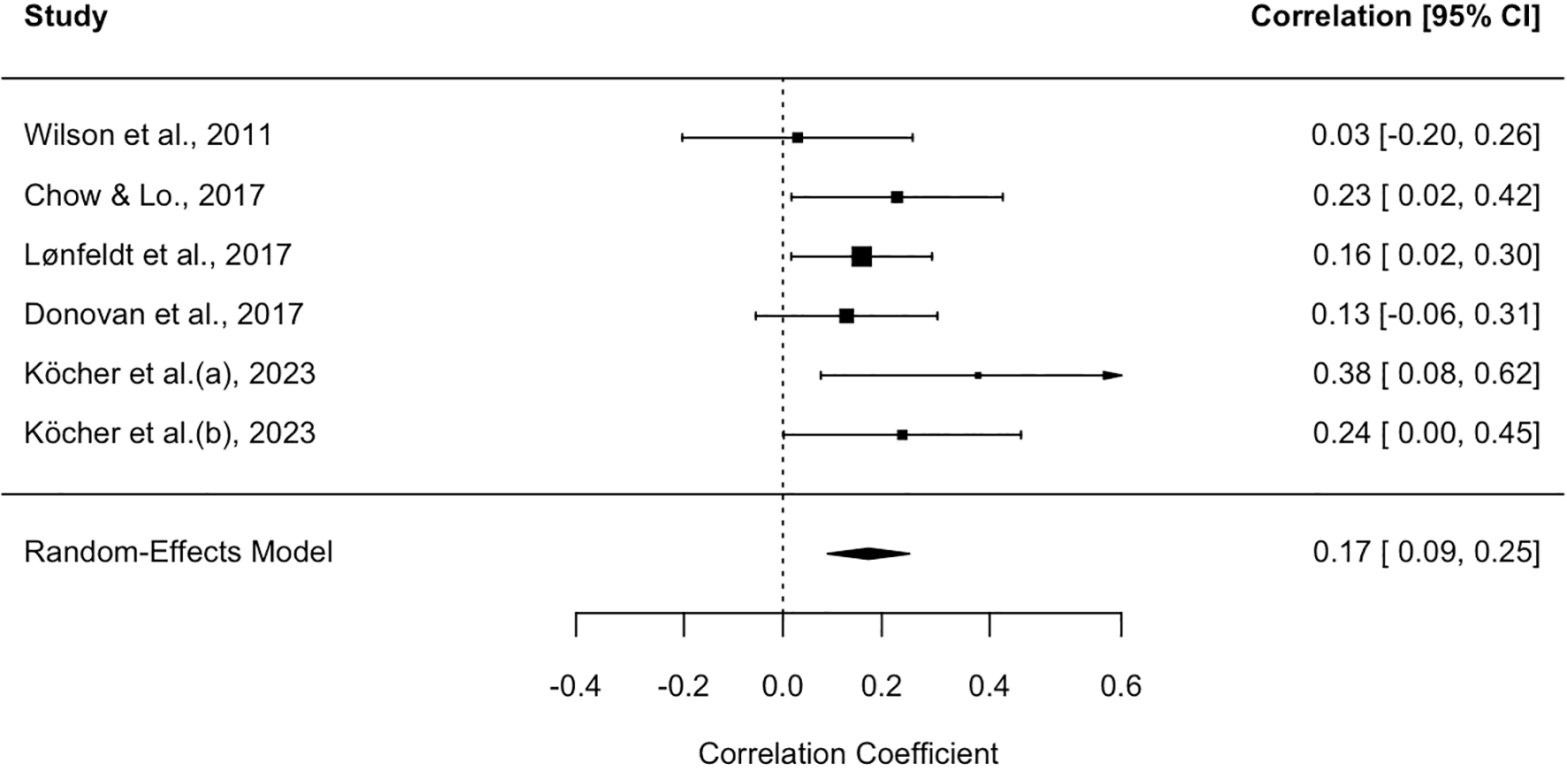
Forest plot for the studies on negative beliefs about danger and uncontrollability of thoughts. Each study included in the meta-analysis is represented by a point estimate, which is bounded by a 95% CI. The summary effect size is displayed as a polygon at the bottom of the plot, with the width of the polygon representing the 95% CI.

The meta-analysis of NEG was conducted on the same group of studies as the previous domain; the results of the corresponding forest plot are presented in **Figure 3**.The random effects model employed revealed a mean effect size of.17 (SE = .04, 95% CI = .09,.26; test of null (2-tail): z-value = 4.11, p <.01, k = 6), suggesting that, in this case as well, there is a relationship between these types of metacognitive beliefs in parents and their offspring. Since heterogeneity was not found between studies (I² = 0%, CI:.00, 86.54; Q = 4.18, p = .52), no further analyses to test the role of possible moderators were conducted.

Regarding publication bias, the funnel plot (**Supplementary Figure S2** in **Supplementary Material**) did not reveal any asymmetry, which is consistent with Egger’s test, which was also not significant in this case (b = .01, 95% CI = -.30,.36, z-value = .97, p = .33).

#### 3.6.3 Cognitive self-consciousness

**Figure 4:**
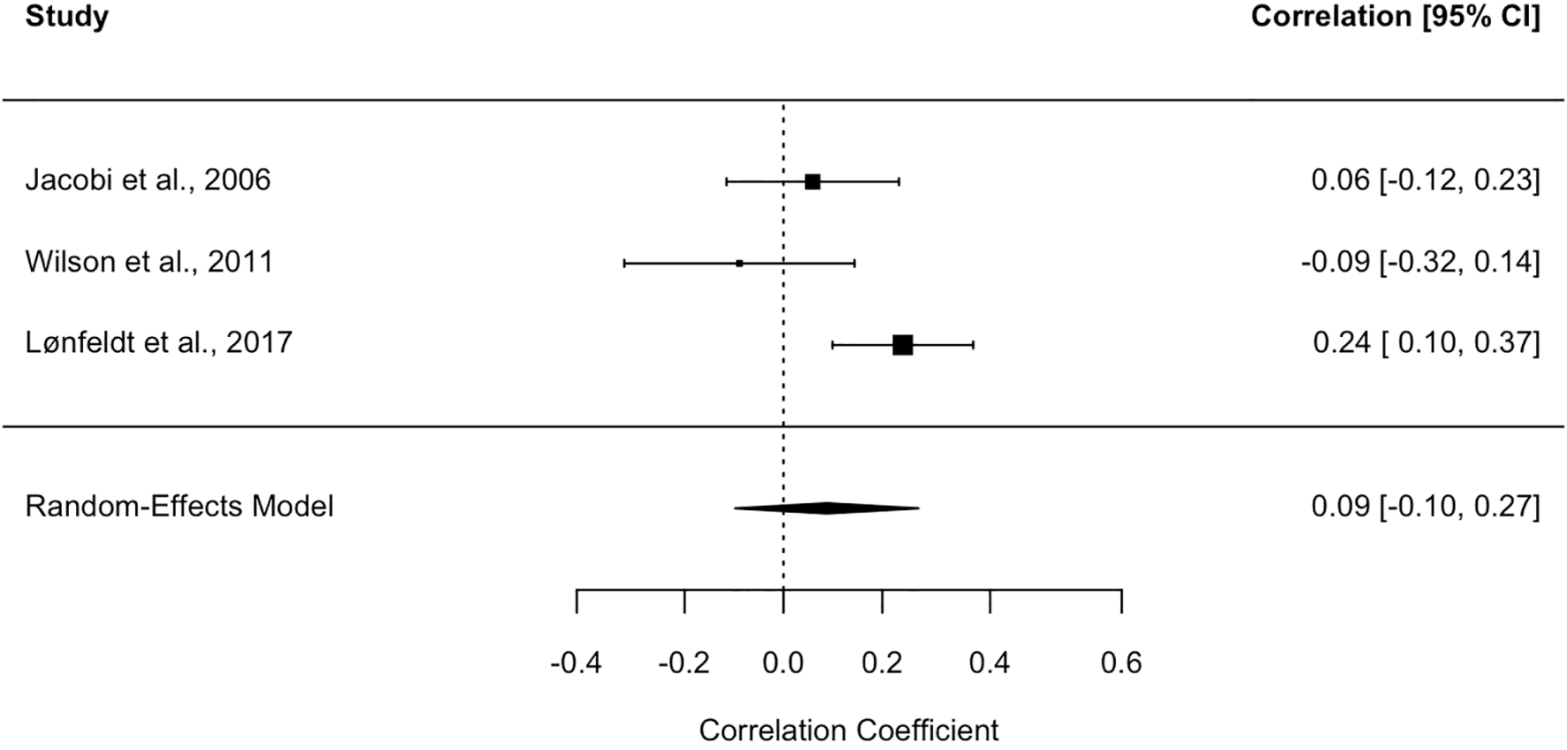
Forest plot for the studies on cognitive-self-consciousness. Each study included in the meta-analysis is represented by a point estimate, which is bounded by a 95% CI. The summary effect size is displayed as a polygon at the bottom of the plot, with the width of the polygon representing the 95% CI.

This domain was investigated in three samples drawn from three studies (60, 63, 64). The random-effects model revealed a pooled correlation coefficient of.09 between this type of metacognitive belief in parents and children (see Forest plot in **Figure 4**), which, however, did not reach statistical significance (SE = .09, 95% CI = –.09,.28; test of null [two-tailed]: z = 0.92, p = .35, k = 3).

By contrast, heterogeneity across studies was significant (I² = 68.63%, CI = .01, 99.22; Q = 6.39, p<.05), prompting further analyses to test the effect of moderators.

Both meta-regressions with the continuous variables “quality scores of the studies” (β = .95, SE = 1.30, 95% CI = –1.60, 3.51, z = .73, p = .47) and “children’s age” (β = –.03, SE = .04, 95% CI = –.11,.05, z = –.75, p = .44) yielded non-significant results.

With respect to potential publication bias, visual inspection of the funnel plot (**Supplementary Figure S3** in the **Supplementary Material**) indicated asymmetry, a conclusion further supported by the significant Egger’s test (b = .76, 95% CI = .24, 1.30, z = –2.45, p <.05).

After correction, the adjusted effect size was found to be significant (adjES = .24, 95% CI = .02,.47, p <.05), confirming that the effect size estimate may also in this case have been influenced by publication bias.

## 4 Discussion

The primary aim of this systematic review and meta-analysis was to provide a solid starting point for future research on the origins of metacognitive beliefs, by analyzing studies that addressed their intergenerational dimension through the investigation of associations with genotype or with parental metacognitive beliefs.

The findings of the literature search clearly indicate that research on this topic remains limited, with few relevant studies identified, particularly concerning the association between metacognitive beliefs and genotype, which remains largely unexplored. A total of nine pertinent articles were retrieved, eight examining associations between parental and offspring metacognitive beliefs, and only one directly investigating the link between a psychometric measure of metacognition and participants’ genotype.

The results presented narratively across the nine included studies were highly heterogeneous, suggesting that the intergenerational dimension might be more pronounced for some metacognitive domains than for others.

The meta-analytic results focusing on the POS and NEG domains suggest that, at least for these two dimensions of metacognitive beliefs, there is a significant small-to-moderate association between parents and children (r = .24 and r = .17, respectively), supporting the hypothesis that part of the origins of metacognitive beliefs may lie in the relationship between children and their family environment, in line with the literature on the development and intergenerational transmission of cognitive biases (30–32).

The larger estimates observed for positive compared to negative beliefs may be partially explained by the fact that POS are more easily verbalized and directly modeled in daily interactions (e.g., statements such as “worrying helps me prepare”) (28, 33), whereas NEG may be more internalized and less likely to be openly communicated by parents (33, 77). In line with this, Köcher et al. (62) reported stronger parent-child associations for both positive and negative beliefs in non-clinical samples compared to clinical ones, suggesting that the chronicity or severity of psychopathology may disrupt typical familial interactions, possibly due to broader cognitive or emotional dysregulation interfering with intersubjective learning (78, 79). Further support for this interpretation comes from studies showing a relationship between parental interaction styles and children’s metacognitive beliefs, specifically highlighting associations between increases in children’s dysfunctional metacognitive beliefs and parenting styles characterized by harshness or overprotection (33, 77, 80).

Regarding the CSC domain, the results were mixed, but overall, the meta-analysis of the three studies including this domain did not reveal a significant parent–child association. An intriguing hypothesis is that this association may be absent because CSC reflects a more elaborated and pervasive form of metacognition, potentially emerging as a consequence of the application of earlier metacognitive beliefs that consolidate the CAS and lead to subsequent thought hyper-monitoring. This hypothesis is consistent with findings showing that self-awareness and self-reflectiveness tend to become more accurate with age (81), The CSC scale may thus reflect a domain of metacognitive beliefs less shaped by family influences in childhood and early adolescence, and more by individual experiences and biological predispositions.

However, this consideration is not supported by the findings of Fekih-Romdhane et al. (61), who did not observe a significant association for the CSC domain.

Nevertheless, although the findings of this study should be interpreted with caution, as they were based on a clinical sample of individuals with schizophrenia and are therefore not representative of the general population, they provided preliminary evidence for associations of CC and NC with genotype.

Specifically, the authors report a significant association between these two metacognitive domains and the COMT rs4680 polymorphism, a gene implicated in dopaminergic regulation and prefrontal functioning. These findings appear to align with preliminary results observed for the DRD4 gene. Certain allelic variants of COMT rs4680, in fact, lead to increased dopamine degradation in the prefrontal cortex, which can result in deficits in executive functions such as working memory and attention, elements that, as discussed, play a central role in the metacognitive model (61, 82).

### 4.1 Limitations

When interpreting the results of the present study, some limitations should be taken into account. First, almost all the studies were conducted in Western, educated, industrialized, rich, and democratic (WEIRD) populations, limiting the generalizability of findings to other cultural contexts in which beliefs about emotion and cognition may differ substantially. Second, methodological heterogeneity across studies also warrants caution in interpretation. The issue of heterogeneity across studies is evident from the meta-analytic results, where substantial variance was detected in two out of the three domains. Unfortunately, we were unable to identify factors that could account for this heterogeneity through the selected moderators, whose effects proved statistically non-significant. This outcome may be somewhat unexpected, as it is reasonable to hypothesize that age, in particular, could significantly influence metacognition-related scores and thus contribute to variance across studies. Indeed, metacognitive processes, as theorized by Wells and Matthews (4, 5), involve executive functions, which are known to undergo marked changes during the transition from childhood to adolescence (83).

However, the very limited number of studies suggests that the failure to detect moderation effects is more likely attributable to a statistical limitation, as noted by Higgins et al. (47), rather than to an inappropriate initial choice of moderators.

Third, for both the POS and CSC domains, the funnel plot revealed asymmetry, suggesting potential publication bias. Application of the trim-and-fill method substantially altered the results: in the meta-analysis with seven studies, the initially significant effect became non-significant, albeit marginally (p = .09), whereas in the meta-analysis with three studies, a previously non-significant effect turned significant. These seemingly contradictory findings highlight both the fragility of estimates derived from such a limited number of studies and the inherent limitations of publication bias correction methods (47). This represents another factor that calls for considerable caution in interpreting these findings.

Fourth, although most studies used the Metacognitions Questionnaire (MCQ-30; 41), the specific subscales employed differed, and some studies relied on alternative instruments such as the Positive and Negative Beliefs About Rumination Scale (PBRS/NBRS; 33) or the Cognitive Self-Consciousness Scale-Expanded (CSC-E; 61), potentially influencing the effect size estimates. Moreover, with regard to the instruments used, many of the included studies involved samples in which at least part of the participants were older than 12 years. Recent evidence has shown that for individuals aged 12 to 18, the MCQ-A is preferable for assessing metacognitive beliefs, whereas the MCQ-C is more appropriate for younger children (84). However, none of the studies in question applied this guideline, likely reducing the sensitivity of metacognitive assessment for this subgroup.

Fifth, another important limitation concerns the lack of consistent control for potentially confounding variables across studies, such as child temperament, socioeconomic status, parental psychopathology, or family functioning, which may influence both parental and child metacognitive patterns, as well as exposure to early adversities, which the literature has shown to be associated with dysfunctional metacognitive thinking, particularly NEG (85).

Sixth, eight out of the nine included studies were cross-sectional, limiting the possibility of drawing conclusions about a direct causal link between parents’ and children’s metacognitions.

Seventh, most studies included in this review focused on mother-child dyads, with only limited data available regarding fathers. This maternal bias, also observed in previous developmental psychopathology research, may overlook important paternal influences and dyadic dynamics. Future work should explore whether fathers’ metacognitive beliefs exert comparable effects, or whether differences in parenting style and emotional expressivity moderate the transmission process. Moreover, none of the included studies examined whether gender differences might have a direct effect on the associations detected through the MCQ. Although the literature suggests that gender may not exert a strong impact on the various MCQ domains (84), it is also well established that females are more prone to emotional disorders (86), and this vulnerability could bidirectionally influence the relationship with their parents.

Finally, the presence of only two studies including a clinical sample had a twofold negative impact. On one hand, it precludes generalization of the findings to the clinical population, which is particularly relevant regarding the association with genotype, as the only study testing this was conducted in a clinical sample. On the other hand, it prevented us from testing our second hypothesis, as a subgroup analysis, which could have likely revealed different correlational patterns, could not be performed.

### 4.2 Future directions

The findings of the present work, together with its limitations, may be of value in inspiring future research on this topic. First, although mixed, the results seem to suggest that not all metacognitive beliefs are associated with those of parents, and that some may instead be more strongly linked to a biological predisposition. This heterogeneity highlights the importance of considering all metacognitive domains in future investigations of etiological factors, rather than focusing exclusively on POS and NEG, as many of the included studies did, even though these are the domains most closely related to the maintenance of the CAS in the S-REF model.

With regard to the mechanisms underlying the association between metacognitive beliefs in parents and children, only a few of the included studies attempted to trace the origins of this relationship, all of which were framed within the parent–child relationship and thus attributed to the family environment.

However, research in behavioral genetics has shown that many of these environmental factors may also reflect underlying genetic influences. For example, personality traits such as neuroticism, as well as components of the CAS or even parenting styles, show substantial heritability, predisposing individuals to interact with their environment and with others in specific ways, including within the family context (38, 39, 87).

This gives rise to a specific mechanism known as passive gene–environment correlation, which refers to the association between the inherited genotype and the family environment in which the child is raised (88).

Similarly, a child’s genetically inherited vulnerabilities may in turn shape interactions within the family context, through a mechanism referred to as evocative gene–environment correlation (88).

On this basis, it is conceivable that children’s metacognitive beliefs may also exert a direct influence on those of their parents. Such associations, however, are notoriously difficult to detect. Twin studies represent a particularly promising approach for investigating etiological factors in this field, especially in their extended version, which includes not only twins but also parents. Extended twin family studies make it possible to disentangle genetic and environmental determinants of psychological traits, and further partition shared environmental covariance into that shared only between siblings and that shared by all family members, thereby allowing better control for gene–environment correlation (89, 90).

Another clear indication emerging from our analysis is the pressing need for more longitudinal studies that can provide insight into causal links and allow firmer conclusions on the mechanisms underlying the intergenerational transmission of metacognitive beliefs. To date, only Walczak et al. (29) adopted a longitudinal design, showing that mothers’ metacognitive beliefs predicted those of their children three years later, and that children’s beliefs in turn predicted anxiety symptoms. However, the authors did not identify potential factors driving this transmission. A promising future direction would be to employ longitudinal approaches that simultaneously investigate both genetic and environmental contributions, in order to test whether the association is stable over time and whether its etiological underpinnings remain constant or change across development.

Psychometric measures currently available for the assessment of metacognition, such as the MCQ-30, have demonstrated good temporal stability both in child and adolescent samples and in adult populations (84, 91), making them well-suited for this line of research.

## 5 Conclusion

In sum, this study provides an overview of the existing literature on the intergenerational dimension of metacognitive beliefs. The findings clearly show that this topic remains largely underexplored, given the small number of relevant studies, particularly those examining the relationship between genotype and metacognitive beliefs, which warrants further investigation. The available evidence appears heterogeneous, yet it indicates a small-to-moderate association between parental and offspring metacognitive beliefs, especially for the POS and NEG domains. Conversely, the limited molecular genetics findings suggest that other domains, such as CC and NC, may be more strongly linked to biological predisposition. This study therefore lays the groundwork for future research, highlighting the need for etiological investigations that separately consider the different metacognitive domains and employ research designs capable of disentangling genetic and environmental components, in order to clarify the mechanisms of intergenerational transmission underlying the associations observed here.

## Data Availability

The raw data supporting the conclusions of this article will be made available by the authors, without undue reservation.
