# Supplementary figures and images for: Exploring intergenerational links and genetic correlates of metacognitive beliefs: A systematic review and meta-analysis
Source: Front Psychiatry. 2025 Nov 7;16:1674793. doi: 10.3389/fpsyt.2025.1674793 (PMC12636001; doi:10.3389/fpsyt.2025.1674793)

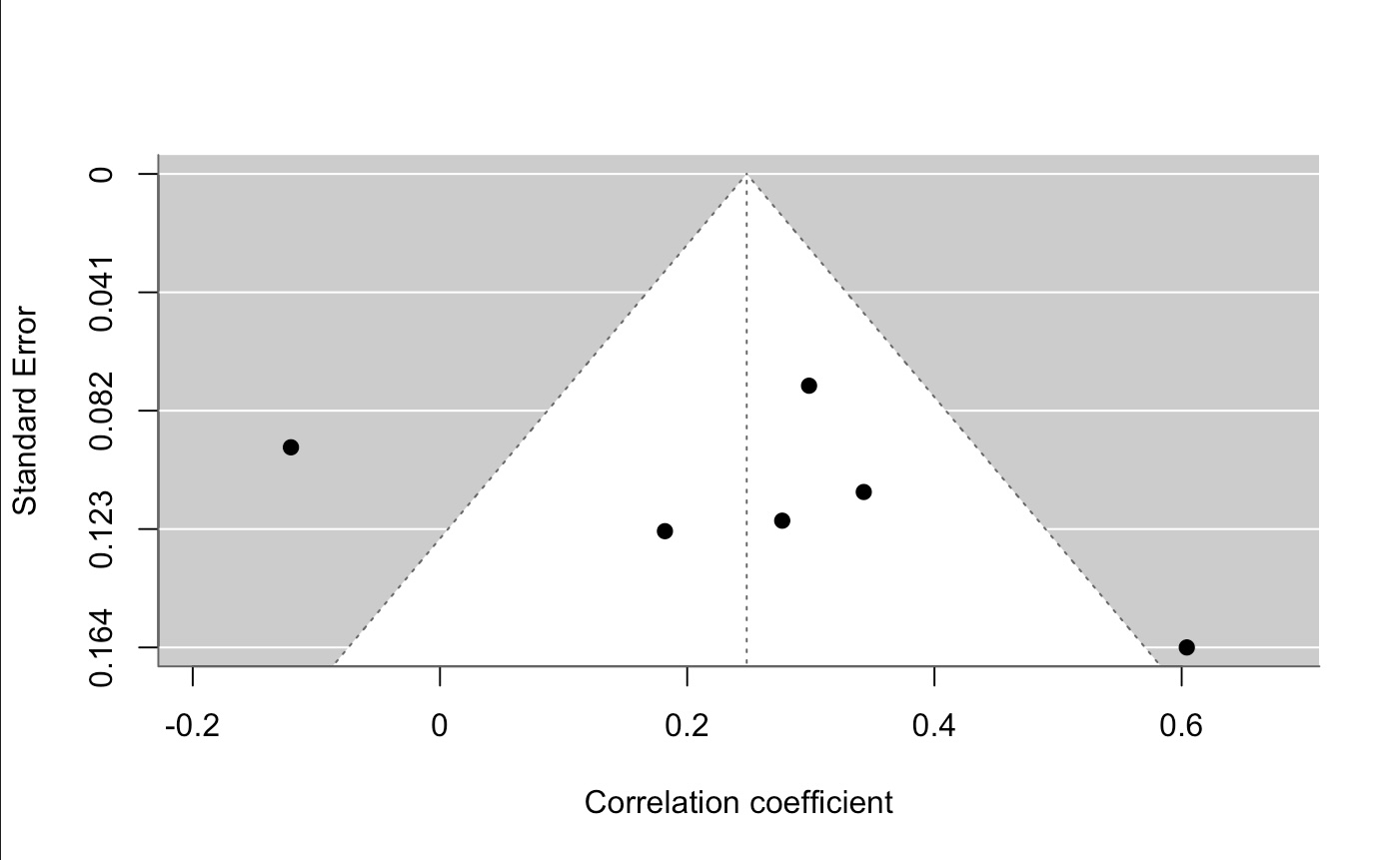

Supplement: Supplementary file 1 [file Image1.jpeg]

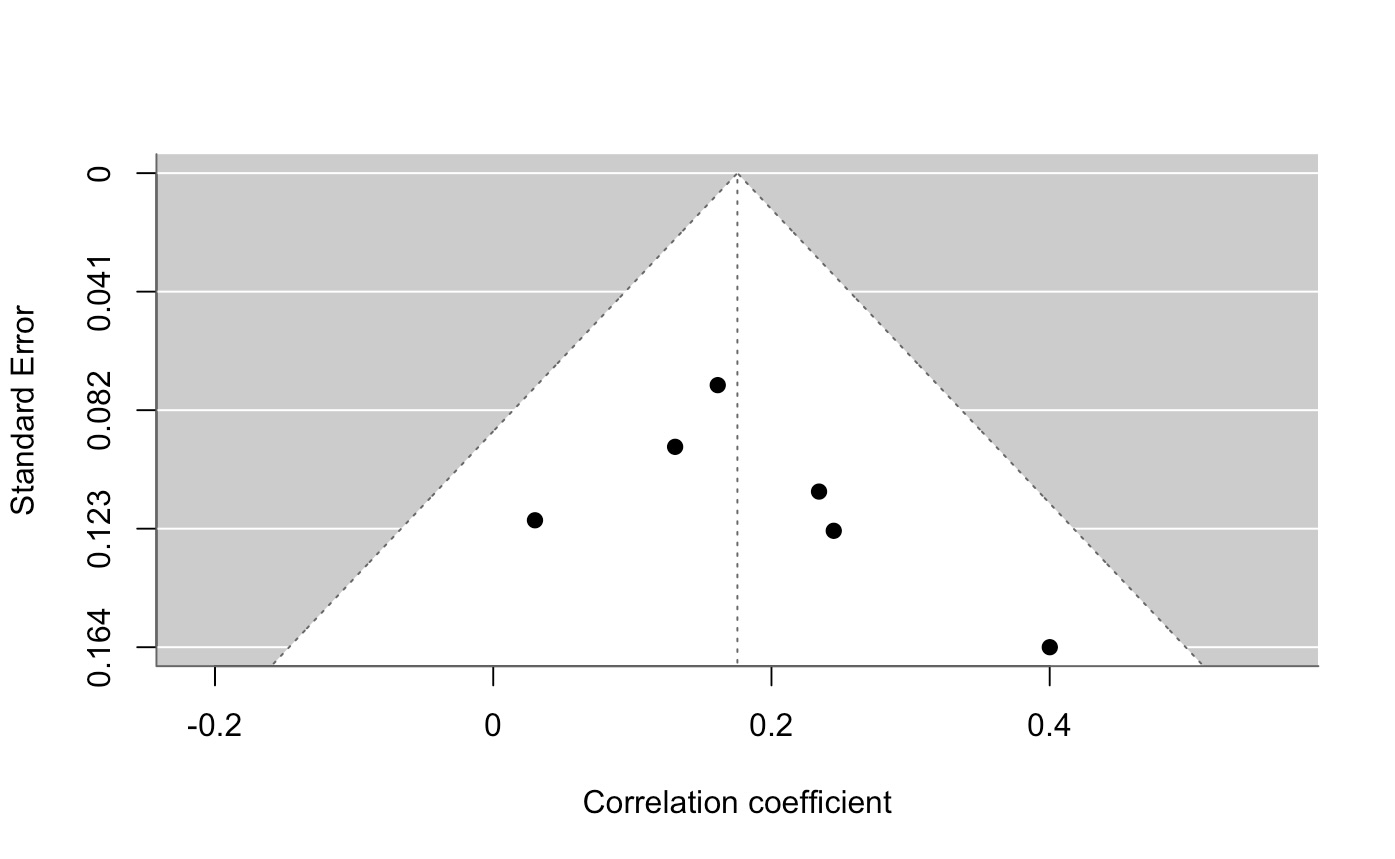

Supplement: Supplementary file 2 [file Image2.jpeg]

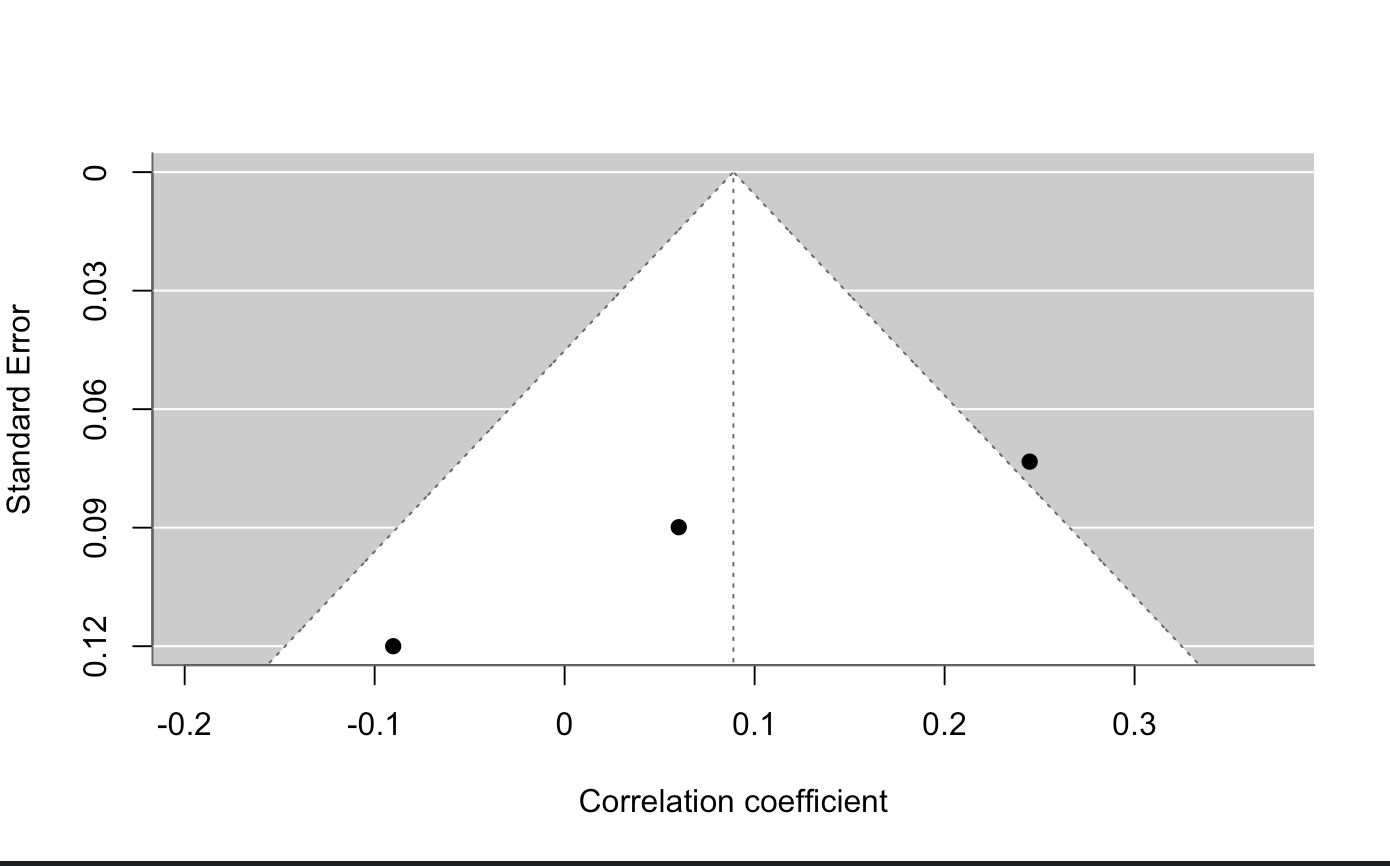

Supplement: Supplementary file 3 [file Image3.jpeg]
